# miR-514a-3p: a novel SHP-2 regulatory miRNA that modulates human cytotrophoblast proliferation

**DOI:** 10.1530/JME-21-0175

**Published:** 2021-11-18

**Authors:** Rachel C Quilang, Sylvia Lui, Karen Forbes

**Affiliations:** 1 Leeds Institute of Cardiovascular and Metabolic Medicine, Faculty of Medicine and Health, University of Leeds, Leeds, UK; 2 Maternal and Fetal Health Research Centre, Division of Developmental Biology and Medicine, Faculty of Biology, Medicine and Health, University of Manchester, Manchester, UK; 3 St. Mary’s Hospital, Central Manchester University Hospitals NHS Foundation Trust, Manchester Academic Health Science Centre, Manchester, UK

**Keywords:** SHP-2, *PTPN11*, miRNA, placenta, trophoblast, proliferation, miR-514, miR-758, IGF

## Abstract

Src homology-2 domain-containing protein tyrosine phosphatase 2 (SHP-2), encoded by the *PTPN11* gene, forms a central component of multiple signalling pathways and is required for insulin-like growth factor (IGF)-induced placental growth. Altered expression of SHP-2 is associated with aberrant placental and fetal growth indicating that drugs modulating SHP-2 expression may improve adverse pregnancy outcome associated with altered placental growth. We have previously demonstrated that placental *PTPN11*/SHP-2 expression is controlled by miRNAs. SHP-2 regulatory miRNAs may have therapeutic potential; however, the individual miRNA(s) that regulate SHP-2 expression in the placenta remain to be established. We performed *in silico* analysis of 3’UTR target prediction databases to identify libraries of Hela cells transfected with individual miRNA mimetics, enriched in potential SHP-2 regulatory miRNAs. Analysis of *PTPN11* levels by quantitative (q) PCR revealed that miR-758-3p increased, while miR-514a-3p reduced *PTPN11* expression. The expression of miR-514a-3p and miR-758-3p within the human placenta was confirmed by qPCR; miR-514a-3p (but not miR-758-3p) levels inversely correlated with *PTPN11* expression. To assess the interaction between these miRNAs and *PTPN11*/SHP-2, specific mimetics were transfected into first-trimester human placental explants and then cultured for up to 4 days. Overexpression of miR-514a-3p, but not miR-758-3p, significantly reduced *PTPN11* and SHP-2 expression. microRNA-ribonucleoprotein complex (miRNP)-associated mRNA assays confirmed that this interaction was direct. miR-514a-3p overexpression attenuated IGF-I-induced trophoblast proliferation (BrdU incorporation). miR-758-3p did not alter trophoblast proliferation. These data demonstrate that by modulating SHP-2 expression, miR-514a-3p is a novel regulator of IGF signalling and proliferation in the human placenta and may have therapeutic potential in pregnancies complicated by altered placental growth.

## Introduction

The type-1 insulin-like growth factor (IGF) receptor (IGF1R) signalling pathway is key for normal human placental and fetal development (Forbes *et al.* 2008, Forbes & Westwood 2010). A key component of the IGF1R signalling pathway required for human placental growth is Src homology-2 (SH2) domain-containing protein tyrosine phosphatase 2 (SHP-2) (Forbes *et al.* 2009). SHP-2, encoded by the *PTPN11* gene, is a ubiquitously expressed cytoplasmic tyrosine phosphatase (Adachi *et al.* 1992, Freeman *et al.* 1992, Ahmad *et al.* 1993, Feng *et al.* 1993, Vogel *et al.* 1993) that interacts with phosphorylated proteins in response to growth factor stimuli. As such, SHP-2 is also a central component of many other signalling pathways and has multiple roles in developmental processes including proliferation, differentiation and angiogenesis (Chang *et al.* 2002, Maile & Clemmons 2002, Mannell *et al.* 2008, Mannell & Krotz 2014). Studies in mice first demonstrated that defects in SHP-2 expression in the placenta lead to fetal growth restriction (FGR) and embryonic lethality (Arrandale *et al.* 1996, Qu *et al.* 1997, Chen *et al.* 2000, Saxton *et al.* 2000, Yang *et al.* 2006). Studies also reveal a key role for SHP-2 in the regulation of human fetal growth. Approximately, half of all cases of Noonan syndrome – a developmental disorder associated with FGR and short stature, have mutations in *PTPN11*resulting in defective IGF-I signalling (Limal *et al.* 2006, De Rocca Serra-Nedelec *et al.* 2012, Cessans *et al.* 2016). Other cases of FGR are also attributed to aberrant placental IGF-I signalling (Laviola *et al.* 2005, Dumolt *et al.* 2021); since SHP-2 is a key component of placental IGF-mediated signalling (Forbes *et al.* 2009), it is likely that modulation of SHP-2 levels could improve placental and subsequent fetal growth. Despite the importance of SHP-2 in normal development, the mechanism of transcriptional and post-transcriptional regulation of SHP-2 is still largely unknown. By reducing the levels of Dicer, a key enzyme in the microRNA (miRNA) biogenesis pathway, in human first-trimester villous explants, we have demonstrated that placental SHP-2 expression is controlled by miRNAs (Forbes *et al.* 2012).

Drugs designed to mimic or inhibit miRNAs have shown therapeutic promise in numerous diseases including hepatitis C (miR-122 inhibitor), type-2 diabetes (miR-103/107) and cancer (miR-34 mimic) (Rupaimoole & Slack 2017, Chakraborty *et al.* 2021), and we have previously shown the safe effective delivery of miRNA inhibitors to the placenta in mice and that targeting key placental regulatory miRNAs improves murine placental and fetal growth (Beards *et al.* 2017). We propose that miRNAs may be a key mechanism for the regulation of placental SHP-2 expression. While many miRNAs are predicted to target individual genes, only a few miRNAs, to date, have been shown to directly target SHP-2 in immune cells (Li *et al.* 2007) and in certain cancers (Patel *et al.* 2016, Cai *et al.* 2018, Long *et al.* 2018); none of which have reported roles in human placenta (Liang *et al.* 2007).

In this study, we aimed to identify miRNAs that modulate SHP-2 expression within the human placenta using high-throughput screens and to determine their potential to modulate IGF-induced cytotrophoblast growth.

## Materials and methods

### Human placental cells and tissue

#### Ethical approval

Human placentas were obtained from elective medical or surgical termination of pregnancy during the first trimester (8–12 weeks) of pregnancy. Term placentas from uncomplicated pregnancies (37–42 weeks) were collected within 30 min of vaginal or elective caesarean delivery (Supplementary Table 1, see section on supplementary materials given at the end of this article). The study had local research ethics committee approval (13/NW/0205; 08/H1010/55(+5)), and all tissue was collected from St Mary’s Hospital, Manchester, following written informed consent.

#### Human placental explant culture

As previously described (Farrokhnia *et al.* 2014), normal, late first-trimester (8–12 weeks) placental tissue was dissected under sterile conditions into 2–3 mm^3^ fragments comprised of small cluster of terminal villous branches. Three pieces, selected at random, were transferred into a 1:1 mixture of serum-free Dulbecco’s modified Eagle’s medium (DMEM) and Ham’s F-12 (Lonza, Cambridge, UK) containing 100 U/mL penicillin, 100 μg/mL streptomycin and 2 mM l-glutamine (Gibco). For term tissue, three random areas of each placenta were biopsied, and the chorionic, non-anchoring villi were dissected from these areas and rinsed in sterile PBS, stored in RNALater overnight at 4°C and transferred to −80°C prior to use in experiments to profile *PTPN11* and miRNA expression.

#### Primary human cytotrophoblast and stromal cells

Primary cytotrophoblast and stromal cells were freshly isolated from first-trimester human placenta using the methods previously established (Harris *et al.* 2006, Ingman *et al.* 2010). Briefly, tissue was dissected, weighed and washed carefully in minimum essential media (MEM). Approximately, 6 g of tissue was transferred to 20 mL of MEM containing 0.125% (v/v) trypsin, 1.3 mM EDTA (Invitrogen), and 0.4 mg/mL DNase I, grade I (Sigma) and incubated for 35 min at 37°C, with occasional resuspension to remove trophoblast cells. Cells were collected, and enzymatic digestion was repeated. Remaining tissue fragments were filtered (100 μm) and used for stromal cell isolation. Trophoblast cells were loaded on a Percoll gradient and centrifuged for 30 min at 1800 ***g***, where cytotrophoblast cells were collected from 30 to 45% range. Cells were pelleted, resuspended in DMEM/F-12 supplemented with 10% FBS (Biosera, Maidenhead, UK), penicillin (0.1 U/mL), streptomycin (100 μg/mL) and l-glutamine (2 mM) and cultured on Matrigel-coated flasks. Immunostaining for cytokeratin-7 (an epithelial marker) and vimentin (a mesenchymal marker) (data not shown) demonstrated 95% purity of the isolated cytotrophoblast cells. Stromal cells were isolated from tissue free of trophoblast cells and were washed (3 × 5 min) in DMEM containing 2.5 mg/mL collagenase and 2 mg/mL hyaluronidase and incubated for 3 h at 37°C. Settling under gravity, the suspension was then centrifuged for 20 min at 700 ***g***, and the resulting pellet was resuspended in 3 mL of MEM. Subsequently, the cell pellet was loaded onto 25/60% Percoll and centrifuged for 30 min at 670 ***g**.* The band of stromal cells and aggregates was removed from the Percoll, added to 35 mL of MEM and centrifuged for 15 min at 100 ***g***. The pellet was then resuspended in DMEM supplemented with 10% FBS, penicillin (0.1 U/mL), streptomycin (100 μg/mL) and l-glutamine (2 mM), and cells were seeded onto flasks coated with rat tail collagen (BD Biosciences).

#### Human placental cell lines

BeWo human choriocarcinoma cells (CCL-98, American Type Culture Collection (ATCC) and the immortalized human first-trimester extravillous trophoblast cell line Swan-71 (Straszewski-Chavez *et al.* 2009) were maintained in 1:1 DMEM:Ham’s F-12 supplemented with 10% FBS, penicillin (0.1 U/mL), streptomycin (100 μg/mL) and l-glutamine (2 mM) at 37°C, 5% CO_2_. JAR (ATCC. HTB-144) and JEG-3 (ATCC 92120308) choriocarcinoma cells were cultured in DMEM, containing 10% FBS and penicillin (0.1 U/mL), streptomycin (100 μg/mL)), 2 mM glutamine at 37°C, 5% CO_2_.

### Identification of candidate SHP-2-regulatory miRNAs

To identify candidate miRNAs that regulate SHP-2 expression, we used a reverse genetics approach. Genome-wide miRNA library screening has been used successfully to identify miRNAs that regulate the expression of specific proteins (le Sage *et al.* 2007, Zhong *et al.* 2010); however, since there are currently 1917 mature miRNAs in the human genome (https://www.mirbase.org/summary.shtml?org=hsa), this can be labour intensive and time-consuming. To generate a smaller, focused miRNA library, we first performed a bioinformatics-driven approach using the miRNA:mRNA-predicted interaction programmes miRabel (Quillet *et al.* 2019) PITA (Kertesz *et al.* 2007), miRanda (John *et al.* 2004), SVMicrO (Liu *et al.* 2010) TargetScan (Lewis *et al.* 2005) to identify miRNAs with predicted binding sites in the 3’UTR of *PTPN11*, the gene encoding SHP-2. Predicted SHP-2-regulatory miRNAs present in four of the five prediction programmes were classed as potential *PTPN11*/SHP-2 regulatory miRNAs.

SureFind Transcriptome PCR complete miRNOME-1 mimic arrays (Qiagen) are array panels containing cDNA preparations obtained from HeLa cells that have been transfected with 90 different specific miRNA mimics to simulate the overexpression of the mature miRNAs and six experimental controls which include non-targeting miRNA mimics. We searched all commercially available SureFind transcriptome (PCR) complete miRNOME mimic array panels to identify an array panel enriched with mimics predicted to bind to *PTPN11*. RT^2^ SYBR Green quantitative (q)PCR mastermix containing *PTPN11-*specific primers (200 nM) (forward, 5′-CCCACAATCAAGATTCAGAACACT-3′; reverse, 5′GCCCGTGATGTTCCATGTAA-3′) or 18S rRNA-specific primers (forward, 5’GCTGGAATTACCGCGGCT-3’; reverse 5’-CGGCTACCACATCCAAGGAA-3’) were applied to the complete miRNome-3 mimic SureFIND transcriptome PCR array (product code: TCMB-303A-1; Qiagen), and qPCR was performed using a Stratagene MX3005P thermal cycler (Agilent Technologies). Data analysis was performed using Qiagen RT^2^ Profiler PCR Array Data Analysis Webportal which used the ΔΔC_T_ method with normalization of the raw *PTPN11* expression data to 18S rRNA. Data were expressed as fold change compared to control (non-targeting miRNA mimic). miRNA mimics found to alter *PTPN11* mRNA expression by at least two-fold were defined as candidate SHP-2 regulators. Binding sites for the seed sequences of these miRNAs in the *PTPN11* 3’UTR were confirmed using miRANDA target prediction database (Betel *et al.* 2008). Validation of miRNA mimic screens was performed using qPCR and miRNA-specific mimics as described below.

### Quantitative qPCR analysis of miRNA and mRNA expression in human placental cells and tissue

#### miRNA

Levels of candidate SHP-2 regulatory miRNAs were assessed in human first trimester and term placental tissue, primary human placental stromal or cytotrophoblast cells or human placental cells lines (BeWo, JAR, JEG, SWAN-71). Total RNA was extracted from cells or tissue using miRVANA miRNA extraction kits (Ambion). RNA quantity and purity were assessed by Nanodrop. All RNA samples had *A*_260_/*A*_280_values in the range of 1.93–2.08 and *A*_260_/*A*_230_ values in the range 2.05–2.14. Total RNA of 25 ng was reversed transcribed using miRCURY LNA™ Universal cDNA Synthesis kit (Exiqon). Individual miRNAs were detected using the miRCURY LNA™ Universal RT microRNA PCR system (Exiqon) and LNA-enhanced miRNA-specific primer sets (Exiqon) for hsa-miR-514a-3p (target sequence: AUUGACACUUCUGUGAGUAGA) and hsa-miR-758-3p (target sequence: UUUGUGACCUGGUCCACUAACC). All reactions were performed in duplicate using Stratagene MX3005P thermal cycler (Agilent Technologies) and 60°C annealing temperature. 5SrRNA was unaffected by treatment; therefore, the relative amount of miRNA was normalized to 5SrRNA. Fold changes in miRNA expression from control (mean of all Δ*Ct* values) were calculated as 2^(−ΔΔ^*^Ct^*^)^, where ΔΔ*Ct* = Δ*Ct* control − Δ*Ct* treated sample and Δ*Ct* = *Ct*_miRNA_ − *Ct*_5S__rRNA_.

#### mRNA

Levels of *PTPN11* were assessed in human first trimester and term placental tissue. Total RNA of 100ng was reverse transcribed using AffinityScript Multi-Temperature cDNA synthesis kit (Agilent). cDNA was diluted 1:10, and qPCR was performed using Brilliant III Ultra-Fast SYBR Green qPCR Master Mix (Agilent) and specific primers for *PTPN11* (forward, 5′-CCCACAATCAAGATTCAGAACACT-3′; reverse, 5′GCCCGTGATGTTCCATGTAA-3′) and 18S rRNA-specific primers (forward, 5’-GCTGGAATTACCGCGGCT-3’; reverse 5’-CGGCTACCACATCCAAGGAA-3’. 18S rRNA was unaffected by treatment; therefore, the relative amount of mRNA was normalized to 18S rRNA.

### Transfection of pre-miRNA mimetics and SHP-2 siRNA in first-trimester human placental explants

Pre-miR™ miRNA precursors specific for hsa-miR-514a-3p (200 nM; target sequence: AUUGACACUUCUGUGAGUAGA), hsa-miR-758-3p (200 nM; target sequence: UUUGUGACCUGGUCCACUAACC), Cy™ 3-labelled pre-miRNA precursor negative control (Pre-miR-C; 200 nM; Ambion ), short interfering RNA (siRNA) targeting *PTPN11* (UAGUGUUUCAAUAUAAUGCUGGACC; 100 nM) or non-targeting siRNA (100 nM; NT; Ambion) were transfected into first-trimester human placenta explants using Nucleofector programme X-005 (Amaxa Biosystems, Germany) using basic primary mammalian epithelial cell nucleofector solution as previously described (Forbes *et al.* 2009). Following transfection, explants were maintained in culture for 48–72 h on 1% agarose coated 24-well plates as previously described (Farrokhnia *et al.* 2014). Overexpression of the miRNAs was confirmed by qPCR using specific primers as described above. The effect of the siRNA or miRNA sequences was compared to that of three controls: untreated tissue (control), tissue exposed to the transfection procedure alone (mock) or transfected with non-targeting siRNA or pre-miRNA precursor negative control. These controls formed the reference for the evaluation of the effect of the siRNA and pre-miRNA precursors on downstream protein expression and cell function.

### Analysis of SHP-2 protein expression

#### Immunohistochemistry

First-trimester explants exposed to miRNA mimetics for up to 48 h were fixed in 4% paraformaldehyde and processed for immunohistochemistry as previously described (Forbes *et al.* 2008). Sections were boiled in 0.1 M sodium citrate buffer to maximize antigen retrieval. SHP-2 localization was examined using rabbit polyclonal anti-human SHP-2 antibody (1:50; Cell Signaling Technologies) followed by a biotinylated swine anti-rabbit IgG antibody (1:200; DakoCytomation Ltd.). Staining was visualized using the avidin-peroxidase method with haemotoxylin counterstain as previously described (Forbes *et al.* 2008).

#### Western blotting

To confirm the effect of the miRNA overexpression on protein expression, 72 h post-transfection first-trimester placental explant lysates were prepared in radioimmunoprecipitation assay (RIPA) buffer as previously described (Forbes *et al.* 2008). Protein of 50 μg from each sample was resolved by SDS-PAGE and transferred to nitrocellulose membranes for Western blotting with antiserum specific for SHP-2 (rabbit polyclonal; 1:5000, Santa Cruz Biotechnology Inc.). Immune complexes were visualized by probing with HRP-conjugated goat anti-rabbit-IgG antibody (1:2000; Dako UK ) followed by chemiluminescence reagents and exposure to x-ray film. ImageJ software was used to quantitate bands corresponding to SHP-2. Membranes were stripped (2% SDS, 100 mM β-mercaptoethanol, 50 mM Tris, pH 6.8, for 30 min at 50°C) and re-probed with an antibody specific for either SHP-1 (1:5000, Santa Cruz Biotechnology Inc.) – to confirm that SHP-2 siRNA specifically altered SHP-2 protein expression and not that of SHP-1 which has the high sequence homology to SHP-2, or for β-actin (1:1000; Sigma clone AC-15) to confirm equal protein loading.

### Assessing potential interaction of candidate miRNAs with PTPN11 mRNA by analysing microRNA-ribonucleoprotein complex (miRNP)-associated mRNA

Mature miRNAs are guided to 3’UTRs of their target genes following association with ribonucleoproteins in the RNA-inducing silencing complex (RISC). The RISC complex contains a number of proteins, including members of the Agonaute (Ago) family. Antibodies to Agonaute proteins are commercially available and allow immunoprecipitation of the RISC complex (ribonucleoprotein immunoprecipitation/RIP). This method can be used to identify miRNA targets since isolated Ago immunocomplexes also contain functional RISC–miRNA–mRNA complexes (Easow *et al.* 2007, Wang *et al.* 2010). By comparing the microRNA ribonucleoprotein (miRNP)-associated mRNAs from control and miRNA-overexpressing cells or tissue, miRNAs that directly interact with specific mRNAs can be identified. First-trimester placental explants were exposed to miRNA-mimetics (200 nM) for 24 h, and miRNP–mRNA complexes were isolated using the RIP-assay kit for microRNA following the manufacturer’s instructions (MBL International Corporation, Massachusetts, USA). Briefly, explants were lysed in mi-lysis buffer and pre-cleared by incubation with protein G beads. Pre-cleared lysates of 500 mg were then applied to Ago-2 antibody (15 mg) immobilized protein-A beads (30 mL) and incubated overnight at 4°C. Beads were washed, and lysates were collected for Western blotting and for subsequent RNA extraction using miRVANA miRNA isolation kit (Ambion). Western blotting was performed to confirm successful immunoprecipitation of the RISC complex, and qPCR analysis was performed to determine the levels of *PTPN11* mRNA present in the complexes.

### Cell proliferation assays

As previously described, first-trimester placental explants were mock transfected, transfected with siRNA, miRNA-specific mimics or non-targeting miRNA mimics (Farrokhnia *et al.* 2014) and were cultured in serum-free medium for 48 h and then exposed to 5-bromo-2’-deoxyuridine (BrdU; 100 μM) and IGF-I (10 nM), or vehicle and cultured for a further 24 h. At the end of the experiment, placental tissue was fixed, embedded in wax and levels of BrdU-positive trophoblast cells were assessed by immunohistochemistry as previously described (Forbes *et al.* 2008).

The number of immunopositive (proliferating) cells was expressed as a percentage of total cytotrophoblast nuclei.

### Statistical analysis

Data were analysed using GraphPad Prism 6 Version 6.05 (GraphPad Software Inc.). Comparisons between first trimester and term placentas were done using Mann–Whitney *U* test. The correlation between levels of miR-514a-3p and miR-758-3p with *PTPN11* expression was assessed by Spearman rank correlation test. mRNA expression was analysed using the Wilcoxon-signed rank test, and data are presented as median and range mRNA expression relative to the control (non-transfected) sample for the corresponding experiment. Proliferation data presented as median and range were analysed using the Kruskal–Wallis ANOVA followed by Dunn’s *post hoc* test that was used to compare differences between groups. Results were considered significant at *P* < 0.05.

## Results

### miR-514a-3p and miR-758-3p are candidate regulators of SHP-2 expression

**Figure 1:**
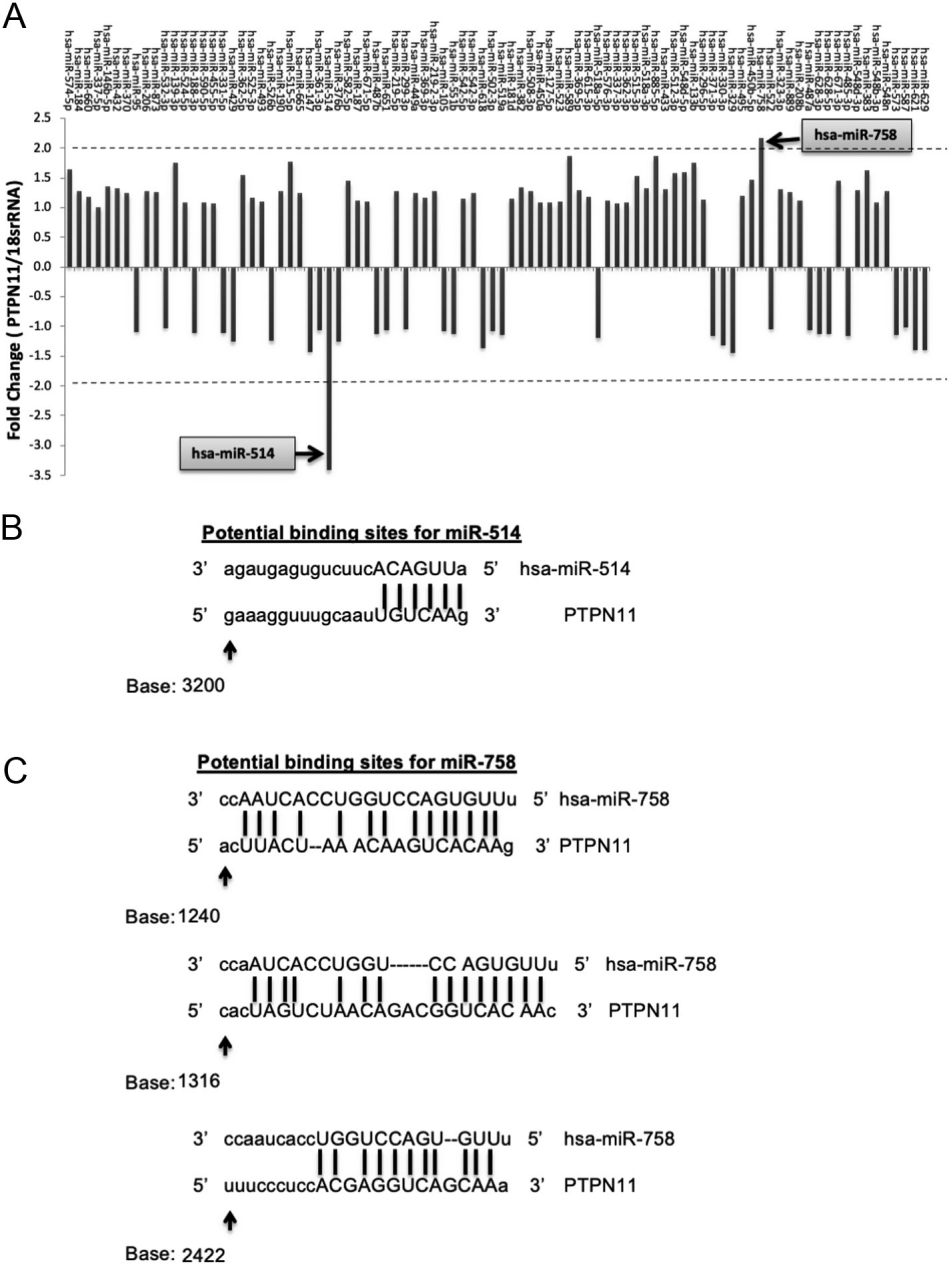
Identification of SHP-2 regulator miRNAs and potential binding sites for SHP-2 mRNA. Primers specific for *PTPN11* were applied to miRNA SureFind transcriptome PCR array containing cDNA from HeLa cells overexpressing 90 different miRNA mimetics and relevant controls. *PTPN11* expression was expressed as fold change in expression relative to 18S rRNA. Samples with a change greater than two-fold were considered potential SHP-2 regulatory miRNAs. *In silico* analysis was performed to determine if the seed sequences of miRNAs that altered *PTPN11*mRNA expression-miR-514a-3p (B) and miR-758-3p (C) had potential binding sites in *PTPN11* 3’UTR. Dashed lines represent consensus binding sites between miRNAs and *PTPN11*. Lower case base letters represent bases with no homology between the individual miRNA and *PTPN11*.

In total, 485 miRNAs were identified as potential PTPN11 regulatory miRNAs (Supplementary Table 2). Samples for 58 of these miRNAs were present in the SureFind transcriptome PCR mimic array, representing 66% of the total mimic samples present on the array (Supplementary Table 3). Despite the array library being enriched for predicted miRNAs that target SHP-2, *PTPN11* expression was only altered by 2 of the 90 miRNA mimics assessed (Fig. 1A and Supplementary Table 3). *PTPN11* expression was reduced by 3.40-fold in samples overexpressing miR-514a-3p and was increased (by 2.16-fold) in samples overexpressing miR-758-3p, suggesting a role for these miRNAs in the regulation of SHP-2 expression. *In silico* analysis using miRANDA target prediction database (Betel *et al.* 2008) demonstrated that there are potential binding sites for both miR-514a-3p and miR-758-3p in the *PTPN11* 3’UTR (Fig. 1B and C). Therefore, both miR-514a-3p and miR-758-3p were selected for subsequent analyses.

### miR-514a-3p negatively correlates with placental SHP-2 expression

**Figure 2:**
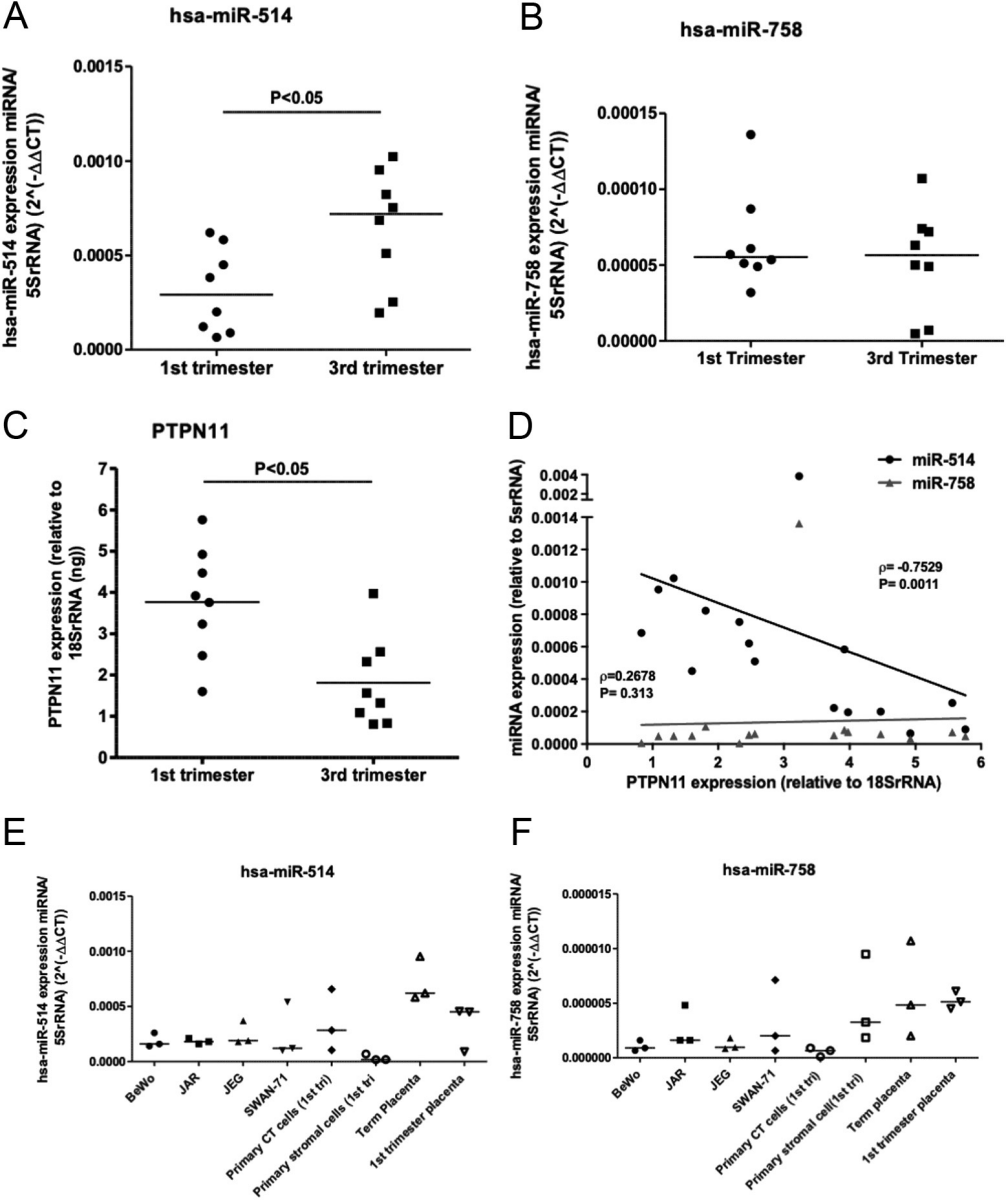
miR-514a-3p and miR-758-3p expression in human placental tissue and cells. Total RNA was isolated from fresh first trimester (*n* = 8) and term (*n* = 8) human placental tissue, and levels of miR-514a-3p (A), miR-758-3p (B) and *PTPN11* mRNA (C) were assessed by qPCR. Data were expressed relative to 5SrRNA, and lines show median relative expression levels. Differences in expression between the two gestational age periods were considered different when *P*< 0.05 (Mann–Whitney *U*-test). The correlation between the levels of miR-514a-3p and miR-758-3p with *PTPN11* expression was assessed by Spearman rank correlation test (D). r and *P*values for each miRNA are displayed. Levels of miR-514a-3p (E) and miR-758-3p (F) were assessed in a panel of placental cell lines (BeWo, JAR, JEG and SWAN-71), primary cells from first-trimester placenta (primary cytotrophoblast, CT and primary stromal cells) and fragments of whole placental tissue from term or first-trimester placenta. Levels are expressed as levels of specific miRNA relative to 5SrRNA.

Further investigations were carried out to determine if the initial library screens in HeLa cell samples (Fig. 1A) translated into the human placenta. qPCR analysis confirmed that miR-514a-3p and miR-758-3p (Fig. 2A and B) were present in both first and third-trimester human placenta. miR-514a-3p was significantly higher in the third trimester than in the first trimester (Fig. 2A; *P*= 0.028; *n*  = 8), but miR-758-3p remained at similar levels throughout pregnancy, albeit in lower levels than miR-514a-3p (Fig. 2B). Expression of *PTPN11* mRNA, the transcript for SHP-2 protein, was highest in the first trimester of pregnancy (Fig. 2C; *P*= 0.010; *n*  = 8). This inversely correlated with levels of miR-514a-3p, but not miR-758-3p, in the placenta (Fig. 2D; *r* = −0.7529, *P*= 0.0011). Furthermore, analysis of miRNA expression in different cell types isolated from the human placenta reveals that similar to SHP-2 (Forbes *et al.* 2009), miR-514a-3p is predominantly expressed in the trophoblast (Fig. 2E). In contrast, levels of miR-758-3p appeared to be higher in the placental stroma (Fig. 2F).

### miR-514a-3p directly regulates SHP-2 expression in the human placenta

**Figure 3:**
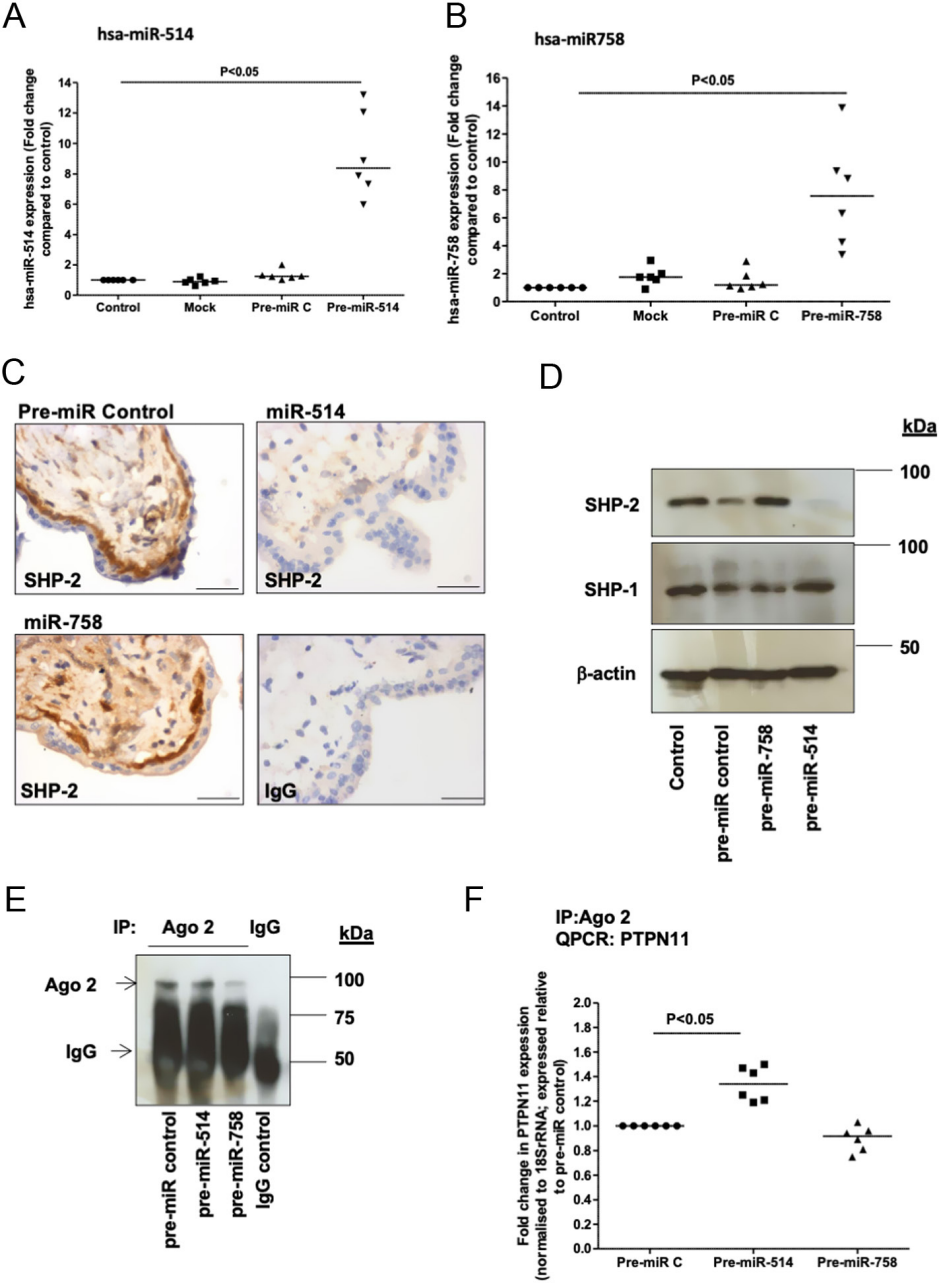
miR-514a-3p regulates SHP-2 expression in the human placenta, but miR-758-3p does not. miR-514a-3p, miR-758-3p or negative control (pre-miR C) mimetics (200 nM) were transfected in to first-trimester human explant tissue for up to 48 h. Overexpression of miR-514a-3p (A) and miR-758-3p (B) was confirmed by qPCR, (Wilcoxon-signed rank test; *n*  = 6; *P*< 0.05). SHP-2 protein expression was examined by immunohistochemistry (C) and Western blotting (D). Blots were stripped and re-probed for β-actin (as an internal control) and SHP-1 (as a control for PTP expression). For immunohistochemistry, rabbit IgG was used as a negative control. Scale bars on images represent 50 μm (C). To determine if miRNAs bound directly to *PTPN11* mRNA, Ago-2-bound miRNA:mRNA complexes were immunoprecipitated (IP) from placental lysates. Recovery of Ago-2 complexes was confirmed by Western blotting (E). Assessment of Ago-2 levels in immunocomplexes recovered using mouse IgG confirmed specificity of the IP. qPCR analysis (F) revealed that levels of *PTPN11* in Ago-bound miRNA:mRNA complexes were significantly increased after the overexpression of miR-514a-3p (Wilcoxon-signed rank test; *n*  = 6; *P*< 0.05).

To explore if miR-514a-3p and miR-758-3p were functional in the placenta, these miRNAs were overexpressed in first-trimester placental explants using specific mimetics (compared to non-transfected, mock-transfected or non-targeting miRNA mimic controls *P*< 0.05; *n*  = 6). miR-514a-3p (Fig. 3A), but not miR-758-3p (Fig. 3B) overexpression, reduced SHP-2 protein expression in the placenta (Fig. 3C and D). qPCR analysis of Ago-bound miRNA:mRNA complexes (Fig. 3E) confirmed that *PTPN11* was present in immunocomplexes under control conditions (Pre-miR C; Fig. 3F). Levels of PTPN11 were significantly higher in the Ago-bound miRNA:mRNA complexes when miR-514a-3p was overexpressed (*P*< 0.05, *n*  = 6). Recovery of *PTPN11* from miRNA complexes was unaffected by miR-758-3p overexpression (Fig. 3F). This demonstrates direct interaction of miR-514a-3p, but not miR-758-3p, to *PTPN11*in the human placenta.

### miR-514a-3p negatively regulates IGF-induced cytotrophoblast proliferation in the first-trimester human placenta

**Figure 4:**
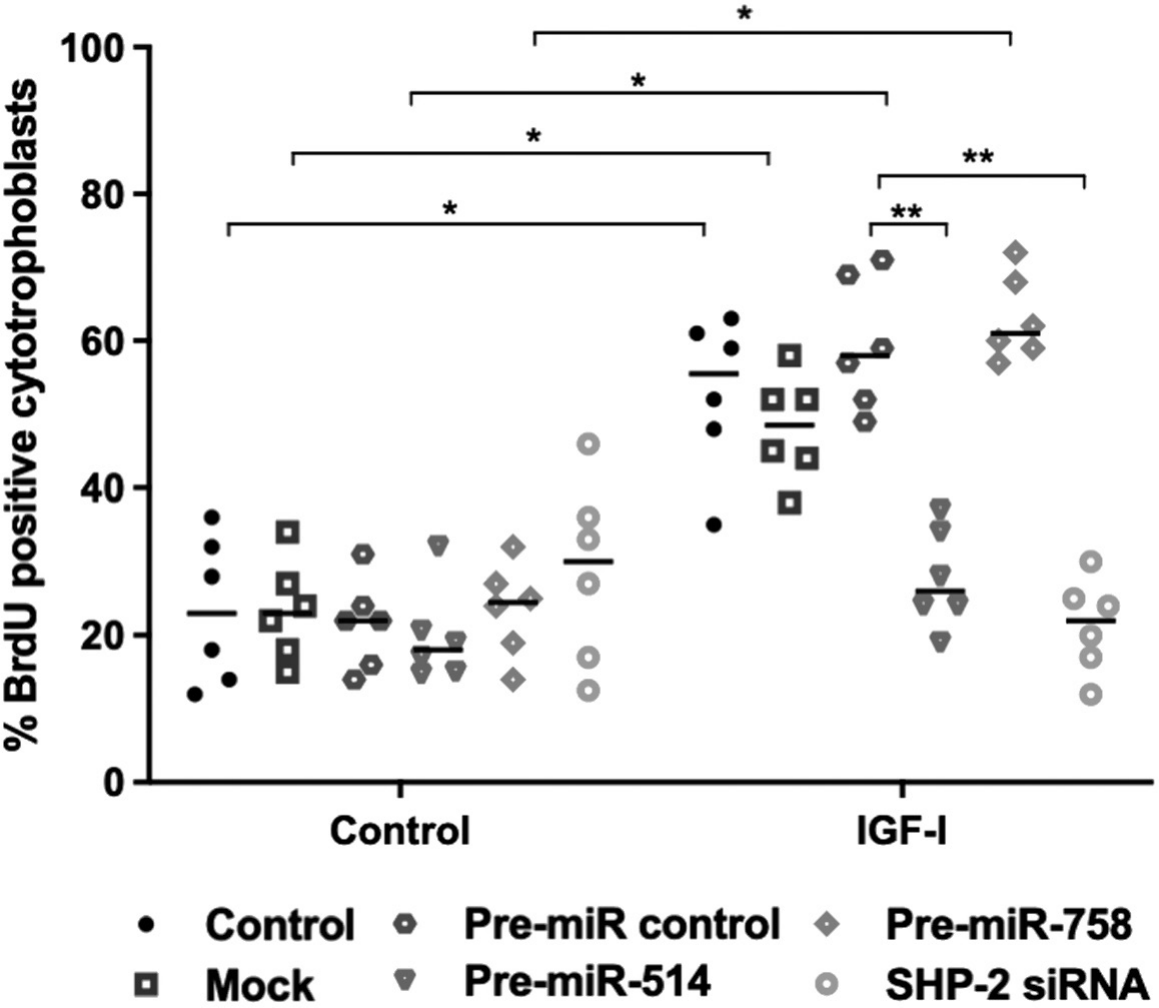
Overexpression of miR-514-a-3p attenuates IGF-induced proliferation in the human placenta. First-trimester placental explants (*n* = 6) were transfected with miR-514a-3p (200 nM), miR-758-3p mimics (200 nM), non-targeting miRNA-mimic (pre-miR-control; 200 nM) or SHP-2 siRNA (100 nM). IGF-I (10 nM) or vehicle was added after 48 h, then 24 h later, cytotrophoblast proliferation was assessed by immunohistochemical analysis of BrdU incorporation and expressed as the number of immunopositive cells as a percentage of total nuclei. Data are presented as median and were analysed by Kruskal–Wallis followed by Dunn’s *post hoc* test. *n*  = 6; *P< 0.05; **P< 0.01.

Under basal conditions, miR-514a-3p overexpression did not alter the levels of cytotrophoblast proliferation (Fig. 4). IGF-I significantly increased cytotrophoblast proliferation compared to vehicle control (Fig. 4; *P*< 0.05; *n*  = 6), and miR-514a-3p overexpression prevented this response (Fig. 4; *P*< 0.01; *n*  = 6). The effect of miR-514 overexpression on IGF-induced cytotrophoblast proliferation was comparable to the effect of SHP-2 siRNA on IGF-induced proliferation. miR-758-3p overexpression, mock transfection and transfection of non-targeting miRNA mimics had no effect on either basal or IGF-I-induced trophoblast proliferation.

## Discussion

It has been previously shown that SHP-2 is an important component of the IGF signalling axis in the placenta (Forbes *et al.* 2009), and miRNAs contribute to the regulation of this signalling and placental growth (Farrokhnia *et al.* 2014). In this study, two miRNAs, miR-758-3p and miR-514a-3p, were identified in HeLa cells as potential SHP-2 regulatory miRNAs, and both miRNAs are expressed in first trimester and term human placenta. By enhancing the expression of miR-514a-3p and miR-758-3p in first-trimester placental explants, it was demonstrated that miR-514a-3p, but not miR-758-3p, directly influences SHP-2 expression and significantly attenuates IGF-I-induced cytotrophoblast growth.

In this study, we took the approach of combining miRNA target prediction algorithms with a miRNA mimic transcriptome library in an attempt to identify specific *PTPN11* regulatory miRNAs. miRNA target prediction algorithms are useful for gaining insights into potential miRNA targets; however, they commonly provide false positives (Riolo *et al.* 2020). We attempted to reduce the number of false positives identified by only classing the miRNAs as potential *PTPN11* regulatory miRNAs if they were predicted by four independent prediction algorithms and experimentally validating these using a miRNA mimic transcriptome array. This high throughput approach is similar to utilizing miRNA mimic libraries. These have been utilized to identify miRNAs for individual genes or for specific cellular functions (Nakano *et al.* 2013, Yarbrough *et al.* 2014, Xiao *et al.* 2015, Martello *et al.* 2018) but are expensive and time-consuming since they require transfections of multiples – sometimes hundreds – of miRNA mimics into cells/tissue prior to functional analysis. miRNA mimic transcriptome arrays overcome these limitations and have been successfully used by other researchers to identify specific gene regulatory miRNAs (Fosbrink 2011, Schaefer *et al.* 2011, Kumar *et al.* 2016, Cerro-Herreros *et al.* 2018). However, these too have their own limitation, for example measuring gene expression, rather than protein to assess miRNA target genes levels, does not take into account that the primary action of many mammalian miRNAs is the repression of protein translation (Ritchie *et al.* 2013). Furthermore, there may be cell-specific miRNA target interactions that are missed by using only HeLa cell cDNA. Indeed, these limitations may explain why, in our study, despite the mimic PCR library being enriched for other predicted miRNAs that target SHP-2, *PTPN11* expression was only altered by two of the miRNA-mimetics analysed. Analysis of SHP-2 protein expression following the overexpression of miRNAs may reveal further candidate SHP-2 regulatory miRNAs identified from target prediction algorithms. Despite the limitations, our combined target prediction algorithm and miRNA mimic transcriptome array approach identified two candidate miRNAs upstream of PTPN11 which we went on to investigate in human placental tissue.

miRNAs usually function as negative regulators of their target genes, however in contrast to this, HeLa cDNA samples overexpressing miR-758-3p showed increased levels of *PTPN11*. It is possible that miR-758-3p is acting indirectly on *PTPN11*/SHP-2 via targeting of interacting partners, or that similar to some other miRNAs, miR-758-3p could be inducing target gene expression by binding to promoter regions of *PTPN11* (Place *et al.* 2008). *In silico* analysis using miRanda target prediction database (Betel *et al.* 2008), however, demonstrated that while there are potential binding sites for miR-758-3p in the *PTPN11* 3’UTR, there are no binding sites for miR-758-3p in the promoter region of *PTPN11*; thus a positive role for miR-758-3p in regulating SHP-2 expression via this mechanism is unlikely. Nonetheless, both miR-514a-3p and miR-758-3p were selected for subsequent analyses.

Reported roles for miR-758-3p include the regulation of proliferation, migration and invasion of carcinoma cells (Wu & Liu 2020, Liu *et al.* 2021, Xiao *et al.* 2021) via modulation of c-Myc and AKT signalling and long non-coding (lincRNA) and circular RNA (circRNA) actions (Ding *et al.* 2021, Xiao *et al.* 2021, Zhang *et al.* 2021). While miR-758-3p do not influence proliferation in the placenta, migration and invasion are also key features of normal placentation (Knofler & Pollheimer 2012), and key roles for lincRNA and circRNA in placental and fetal development are emerging (Gong *et al.* 2021). Thus further studies on these aspects of placental development and function may reveal other roles for miR-758-3p in the placenta.

To our knowledge, there are limited reports in the literature of validated gene targets and functional roles for miR-514a-3p. miR-514a-3p is part of a cluster of 14 miRNAs that collectively have been reported to influence melanoma cell growth and invasion (Streicher *et al.* 2012, Stark *et al.* 2015) and is downregulated in metastatic renal cell carcinoma (Wotschofsky *et al.* 2013); thus, potential roles in the placenta include the regulation of cytotrophoblast proliferation and/or invasion. Arthurs *et al.* (2019) predicted miR-514a-3p to target components of the renin–angiotensin system, specifically downregulating angiotensinogen and angiotensin II type 1 receptor mRNA. Furthermore, miR-514 has been found to be overexpressed in the placentas of women with preeclampsia (Wang *et al.* 2018), commonly associated with placental insufficiency. Furthermore, in the setting of testicular germ cell tumour, loss of miR-514a-3p expression increases paternally expressed gene 3, which activates the nuclear factor kappa B pathway and protects germ cells from apoptosis (Ozata *et al.* 2017), thus demonstrating a pro-apoptotic function of miR-514. In this study, we have demonstrated that miR-514a-3p functions to negatively regulate IGF-mediated placental growth by modulating SHP-2 expression. This growth regulatory role for miR-514a-3p is consistent with the proposed oncogenic role for miR-514a-3p and the miR-506-514 cluster (Streicher *et al.* 2012, Wotschofsky *et al.* 2013). We have not explored the role of miR-514a-3p and/or SHP-2 in regulating other events within the placenta, but given the reported roles for both SHP-2 and miR-514a-3p in invasion and apoptosis in cancer cells, it will be interesting to investigate whether miR-514a-3p/SHP-2 regulates these events in the placenta and indeed in other tissues.

## Conclusion

By utilizing a combination of *in silico* and molecular techniques, miR-514a-3p has been identified as a novel SHP-2 regulatory miRNA in the human placenta. Similar methodology may be useful for identifying additional miRNAs that regulate other specific proteins and/or cellular processes of interest. Ongoing studies will establish whether alterations in placental SHP-2 and/or miR-514a-3p expression are associated with fetal growth disorders. Ultimately, it is anticipated that miRNA-based approaches can be used as therapeutics for correcting abnormal placental growth and cellular proliferation seen in pathological pregnancies.

## Supplementary Material

Supplementary Table 1. Demographic of term placental tissue

Supplementary Table 2

Supplementary Table 3

## Declaration of interest

The authors declare that there is no conflict of interest that could be perceived as prejudicing the impartiality of the research.

## Funding

This study was funded by a University of Manchester Stepping Stones Fellowship (K F), a Society for Endocrinology Early Career Grant (awarded to K F) and a Medical Research Council New Investigator Grant (REF:MR/R023166/1). The Maternal and Fetal Health Research Centre is supported by funding from Tommy’s the Baby Charity, an Action Research Endowment Fund, and the Greater Manchester Comprehensive Local Research Network.

## Author contribution statement

K F designed the study and secured funding. S L and R Q conducted the experiments and data acquisition. All authors (R Q, S L and K F) contributed to the analysis, discussion and interpretation of the data and manuscript drafting. All authors approved the final version of the manuscript.
