# Supplementary material for: miR-514a-3p: a novel SHP-2 regulatory miRNA that modulates human cytotrophoblast proliferation
Source: J Mol Endocrinol. 2021 Nov 18;68(2):99–110. doi: 10.1530/JME-21-0175 (PMC8789026; doi:10.1530/JME-21-0175)
Supplement: Supplementary Table 1. Demographic of term placental tissue [file supplementary_table_1.pdf]

Supplementary Table 1. Demographic of term placental tissue

	Term placental tissue (n=8)
**Age**<br>(years)	31.5 (22 – 38)
**Ethnicity**<br>Caucasian: Other<br>Number (%)	5:3<br>(62.5%:37.5%)
**Smoker**<br>Number (%)	2 (25%)
**BMI at booking**<br>Kg/m^2	26.0<br>(22.5 – 34.2)
**Gestation at delivery**<br>(days)	272<br>(262 – 284)
**Birthweight**<br>(g)	3560<br>(3094 – 4640)
**Fetal Sex**	Male: 4 (50%)<br>Female 4 (50%)
**IBC**	45.5<br>(24 – 81)
**Parity**	1.63<br>(1-4)
**Mode of delivery**	Normal vaginal delivery: 5 (62.5%)<br>Elective CS: 3 (37.5%)

BMI, body mass index; FGR, fetal growth restriction; IBC, individualised birthweight centile; CS Caesarean section
