# Supplementary material for: miR-514a-3p: a novel SHP-2 regulatory miRNA that modulates human cytotrophoblast proliferation
Source: J Mol Endocrinol. 2021 Nov 18;68(2):99–110. doi: 10.1530/JME-21-0175 (PMC8789026; doi:10.1530/JME-21-0175)
Supplement: Supplementary Table 2 [file supplementary_table_2.pdf]

miRNA ID	miRabel score	PITA	miRanda	SVMicrO	Target Scan	Experimentally validated (ExpVal)	binding site in 5'UTR	binding site in coding region (CDS)
hsa-miR-450b-3p	0.00132043	617	99	1260	586	NO	NO	YES
hsa-miR-378a-3p	0.00143759	207	776	1311	138	NO	NO	NO
hsa-miR-934	0.00226988	305	298	1134	447	NO	NO	NO
hsa-miR-1304-5p	0.00310759	243	179	3836	141	NO	NO	NO
hsa-miR-1305	0.00427742	395	1363	1836	1842	NO	NO	NO
hsa-miR-522-3p	0.00429541	312	585	2731	749	NO	NO	NO
hsa-miR-208b-3p	0.00468538	253	1979	242	603	NO	NO	NO
hsa-miR-1205	0.00483143	624	137	4757	311	NO	NO	NO
hsa-miR-217	0.00504804	299	3185	150	1574	NO	NO	YES
hsa-miR-186-5p	0.00511912	1931	1960	1328	1692	NO	NO	NO
hsa-miR-509-5p	0.00530646	547	332	1798	2813	NO	NO	NO
hsa-miR-208a-3p	0.00537874	532	1980	136	588	NO	NO	NO
hsa-miR-509-3-5p	0.00607859	793	328	1800	2849	NO	NO	NO
hsa-miR-1272	0.00626628	1260	1432	675	1495	NO	NO	YES
hsa-miR-1228-3p	0.0075711	328	1885	433	2487	NO	YES	NO
hsa-miR-561-3p	0.00780227	220	3415	1201	1304	NO	NO	NO
hsa-miR-124-3p	0.00811489	1185	1919	2974	819	YES	NO	NO
hsa-miR-873-5p	0.00821033	1215	2137	4115	394	NO	NO	NO
hsa-miR-581	0.00855142	86	1701	1991	1756	NO	NO	NO
hsa-miR-143-3p	0.00883897	3202	893	2085	739	NO	NO	NO
hsa-miR-335-5p	0.00921739	406	2642	1232	2079	NO	NO	NO
hsa-miR-582-5p	0.00989024	774	1390	2867	1019	NO	NO	NO
hsa-miR-409-3p	0.0104121	640	503	2734	2757	NO	NO	YES
hsa-miR-545-3p	0.010688	1509	533	3280	1047	NO	NO	NO
hsa-miR-374b-5p	0.0107144	710	1788	1929	2118	NO	NO	NO
hsa-miR-758-3p	0.0109835	703	1457	2494	1043	NO	NO	NO
hsa-miR-655-3p	0.0113064	2092	1295	1470	1183	NO	NO	YES
hsa-miR-216b-5p	0.0115288	738	1577	2809	1075	NO	NO	NO
hsa-miR-1288-3p	0.0115885	141	2425	2838	657	NO	NO	YES
hsa-miR-129-5p	0.0120618	96	3116	4264	1279	NO	NO	NO
hsa-miR-657	0.0122207	1432	1210	3162	1383	NO	NO	YES
hsa-miR-892b	0.0124408	666	1988	2140	1088	NO	YES	NO
hsa-miR-505-3p	0.01304	547	1443	1662	1473	NO	NO	NO
hsa-miR-362-3p	0.0136861	1873	3069	820	680	NO	NO	NO
hsa-miR-138-5p	0.013946	2741	683	3660	343	NO	NO	YES
hsa-miR-891b	0.0152735	294	2870	897	1427	NO	NO	NO
hsa-miR-298	0.016545201	804	574	4229	3388	NO	NO	YES
hsa-miR-362-5p	0.016837601	3271	407	671	1607	NO	NO	NO
hsa-miR-1286	0.0168918	2981	2037	2032	1359	NO	NO	YES
hsa-miR-548p	0.0170797	1081	4468	1715	1711	NO	NO	NO

hsa-miR-636	0.017554101	646	2063	2735	1633	NO	YES	NO
hsa-miR-1245a	0.017710401	362	2237	2678	1383	NO	NO	NO
hsa-miR-510-5p	0.018373899	3674	158	3363	887	NO	NO	YES
hsa-miR-558	0.0188428	1437	1386	2726	2674	NO	NO	NO
hsa-miR-1243	0.0195345	2322	1167	1959	1830	NO	NO	NO
hsa-miR-338-5p	0.020249	1745	2669	444	2592	NO	NO	YES
hsa-miR-599	0.020559501	526	5105	1554	860	NO	NO	NO
hsa-miR-501-5p	0.0214845	1493	2727	1959	1764	NO	YES	NO
hsa-miR-23a-3p	0.0234324	1691	1923	3165	1949	YES	NO	NO
hsa-miR-28-3p	0.023442	874	2676	1775	728	NO	NO	YES
hsa-miR-223-3p	0.023655999	24	4470	906	1948	NO	NO	NO
hsa-miR-498	0.024004901	269	3696	3295	1955	NO	NO	YES
hsa-miR-374a-5p	0.024374099	2134	2002	2308	1959	NO	NO	NO
hsa-miR-548b-5p	0.024625201	1484	4962	1835	1723	NO	NO	YES
hsa-miR-383-5p	0.0267397	2521	1030	1766	2681	NO	NO	NO
hsa-miR-653-5p	0.0268798	1038	3764	2263	2149	NO	NO	NO
hsa-miR-485-5p	0.026946301	436	4293	4837	897	NO	YES	NO
hsa-miR-99a-5p	0.028137	617	863	530	188	NO	NO	NO
hsa-miR-921	0.028470799	699	2657	2063	465	NO	NO	YES
hsa-miR-218-5p	0.0285636	1298	3805	2020	1639	YES	NO	NO
hsa-miR-556-3p	0.028608499	2381	1497	1201	1435	NO	NO	NO
hsa-miR-942-5p	0.029321801	1140	6295	439	583	YES	NO	YES
hsa-miR-628-5p	0.0306086	2227	173	2071	2222	NO	NO	NO
hsa-miR-578	0.031941801	1047	6116	409	2857	NO	NO	YES
hsa-miR-99b-5p	0.0325846	730	821	541	205	NO	NO	NO
hsa-miR-183-5p	0.033430401	2962	2931	356	3382	YES	NO	NO
hsa-miR-1266-5p	0.033883899	79	3559	4885	2544	NO	NO	YES
hsa-miR-1275	0.035075501	766	5248	2260	2533	NO	NO	NO
hsa-miR-345-5p	0.036424499	536	2612	750	1855	NO	NO	YES
hsa-miR-185-5p	0.038206	3891	2968	2783	2254	NO	NO	YES
hsa-miR-1255a	0.038934398	1260	3579	2002	1936	NO	NO	NO
hsa-miR-554	0.038995601	1666	1794	1884	630	NO	NO	NO
hsa-miR-620	0.039069802	2085	5853	196	2058	NO	NO	NO
hsa-miR-23b-3p	0.042318702	3032	1915	3338	1934	NO	NO	YES
hsa-miR-548i	0.043246102	3123	4798	1891	1822	NO	NO	NO
hsa-miR-621	0.0448272	1482	1451	2500	1029	NO	NO	NO
hsa-miR-1255b-5p	0.048762999	1516	3503	2300	1959	NO	NO	NO
hsa-miR-187-3p	0.0488129	1771	1172	1195	544	NO	NO	NO
hsa-miR-9-5p	0.053849	1954	6229	958	2904	NO	NO	YES
hsa-miR-200b-3p	0.053858001	1781	3922	2480	1208	NO	NO	NO
hsa-miR-1321	0.053941999	1555	4512	5333	1759	NO	NO	YES
hsa-miR-377-3p	0.054179501	1108	6533	705	1863	NO	NO	NO
hsa-miR-301a-3p	0.055060498	1802	4069	2553	2259	NO	NO	NO

hsa-miR-1208	0.057885401	158	6809	1878	2616	NO	NO	NO
hsa-miR-548m	0.0599805	1701	4103	3606	2337	NO	NO	NO
hsa-miR-548a-5p	0.060578	1946	4835	4085	1795	NO	NO	NO
hsa-miR-422a	0.061023701	8	789	-	138	NO	NO	NO
hsa-miR-559	0.061084401	3904	4599	2456	1609	NO	NO	NO
hsa-miR-130a-3p	0.061291799	1924	3951	2834	2244	NO	NO	NO
hsa-miR-130b-3p	0.0615515	1584	3951	3198	2286	NO	NO	NO
hsa-miR-920	0.062229499	2678	2421	4256	1967	NO	NO	NO
hsa-miR-1270	0.062916301	2506	5935	1084	2354	NO	NO	YES
hsa-miR-1291	0.063492902	103	3815	4227	2752	NO	NO	YES
hsa-miR-496	0.063863002	2353	3607	1613	2200	NO	NO	NO
hsa-miR-511-5p	0.064907998	2641	4865	3552	1629	NO	NO	YES
hsa-miR-548d-3p	0.067662902	2381	4987	3869	1391	YES	NO	NO
hsa-miR-365a-3p	0.069891401	690	4725	1143	1616	NO	NO	YES
hsa-miR-663b	0.072725303	3255	2968	1617	2394	NO	YES	NO
hsa-miR-146b-3p	0.0734917	2415	3173	4341	1773	NO	YES	NO
hsa-miR-548c-5p	0.075328603	2306	4743	4673	1699	NO	NO	NO
hsa-miR-105-5p	0.078042597	513	744	-	682	NO	NO	NO
hsa-miR-1827	0.078763902	2298	6866	3076	2644	NO	NO	NO
hsa-miR-328-3p	0.080290698	964	4064	3521	2352	NO	NO	NO
hsa-miR-640	0.082244098	2301	4400	1583	1160	NO	NO	NO
hsa-miR-1296-5p	0.082397602	3496	3335	981	1178	NO	YES	NO
hsa-miR-583	0.0830248	3564	3232	4516	2641	NO	NO	NO
hsa-miR-152-3p	0.083462998	1797	3370	4223	2488	NO	NO	YES
hsa-miR-495-3p	0.084692702	1361	7947	102	2679	NO	NO	YES
hsa-miR-1252-5p	0.084729202	226	1516	5087	1006	NO	NO	NO
hsa-miR-515-5p	0.086231001	264	8239	3741	1738	NO	NO	NO
hsa-miR-342-5p	0.0870739	1176	5038	1399	3077	NO	NO	NO
hsa-miR-618	0.0909326	1123	5356	1988	2325	NO	NO	YES
hsa-miR-330-3p	0.091440499	3856	3381	3117	3067	NO	NO	YES
hsa-miR-548j-5p	0.0981775	3583	4723	4301	1675	NO	NO	NO
hsa-miR-194-5p	0.101057	3237	4922	170	2205	NO	NO	NO
hsa-miR-548b-3p	0.102003001	2209	3492	2046	1267	NO	NO	NO
hsa-miR-1287-5p	0.104130998	3256	5173	3052	2357	NO	NO	NO
hsa-miR-134-5p	0.104172997	3426	4772	1014	1681	NO	NO	NO
hsa-miR-524-5p	0.105801001	1333	6778	4603	1985	NO	NO	NO
hsa-miR-200c-3p	0.106614999	2964	3945	2904	1520	NO	NO	NO
hsa-miR-1229-3p	0.106948003	2583	4101	3178	2534	NO	NO	NO
hsa-miR-1293	0.108906001	989	5852	4950	2945	NO	NO	YES
hsa-miR-1299	0.109182	3801	5826	3914	1637	NO	NO	YES
hsa-miR-500a-5p	0.112999	34	1200	1536	-	NO	NO	YES
hsa-miR-548d-5p	0.113309003	3605	4956	4724	1711	NO	NO	NO
hsa-miR-132-3p	0.117353	3001	4373	23	2175	NO	NO	NO

hsa-miR-548c-3p	0.117416002	4331	7993	2895	2113	NO	NO	NO
hsa-miR-224-5p	0.118683003	424	2792	38	-	NO	NO	NO
hsa-miR-605-5p	0.118860997	1084	1191	-	583	NO	NO	YES
hsa-miR-212-3p	0.119539	1915	4291	1120	2179	NO	NO	NO
hsa-miR-548k	0.121051997	158	7494	4091	2223	NO	NO	NO
hsa-miR-147a	0.121389002	947	4791	3414	2022	NO	NO	NO
hsa-miR-412-3p	0.125375003	24	1606	-	1423	NO	NO	YES
hsa-miR-651-5p	0.127456993	331	1678	4970	1146	NO	NO	NO
hsa-miR-654-3p	0.130322993	3143	5115	2207	1853	NO	NO	YES
hsa-miR-542-3p	0.130364999	2512	5844	2661	1989	NO	NO	NO
hsa-miR-582-3p	0.132262006	2328	527	2759	237	NO	NO	NO
hsa-miR-369-3p	0.133454993	2961	4584	2858	2060	NO	NO	NO
hsa-miR-421	0.134507	12	1055	-	2240	NO	NO	NO
hsa-miR-527	0.137979999	1401	922	-	1517	NO	NO	NO
hsa-miR-518a-5p	0.137979999	1401	922	-	1517	NO	NO	NO
hsa-miR-769-3p	0.141161993	2883	92	-	534	NO	NO	YES
hsa-miR-26b-3p	0.141249001	-	1082	2005	259	NO	YES	NO
hsa-miR-520h	0.145468995	2054	7186	1474	3606	NO	NO	NO
hsa-miR-944	0.145849004	76	3071	-	1321	NO	NO	NO
hsa-miR-381-3p	0.148448005	3676	5098	1443	2692	NO	NO	NO
hsa-miR-136-5p	0.151206002	1794	6690	3336	2556	NO	NO	YES
hsa-miR-520g-3p	0.151559994	2495	7249	1242	3612	NO	NO	NO
hsa-miR-10b-3p	0.153538004	-	691	373	2343	NO	NO	NO
hsa-miR-24-3p	0.155282006	581	3016	13995	1355	NO	NO	NO
hsa-miR-146b-5p	0.156370997	2315	5477	3093	2526	NO	NO	NO
hsa-miR-548g-3p	0.157389998	532	2112	-	1765	NO	NO	NO
hsa-miR-196a-5p	0.158451006	1634	1156	-	742	NO	NO	NO
hsa-miR-151a-3p	0.159679994	2420	2741	1601	1805	NO	YES	NO
hsa-miR-431-3p	0.161952004	-	148	1018	10	NO	NO	NO
hsa-miR-361-3p	0.162396997	2260	5439	4413	1677	NO	NO	NO
hsa-miR-29b-2-5p	0.163338006	-	676	741	2822	NO	NO	YES
hsa-miR-635	0.164086998	3028	2821	2796	2753	NO	NO	NO
hsa-miR-376b-3p	0.165677994	208	2578	2808	968	NO	NO	NO
hsa-miR-645	0.170214996	4585	3437	2072	1477	NO	NO	YES
hsa-miR-1184	0.170966998	670	3657	1058	-	NO	NO	YES
hsa-miR-302d-3p	0.171752006	4035	5527	3144	2377	NO	NO	NO
hsa-miR-499a-5p	0.176079005	426	1069	1972	-	NO	NO	NO
hsa-miR-489-3p	0.177898005	2287	980	3540	778	YES	NO	NO
hsa-let-7f-2-3p	0.178652003	-	1794	819	1544	NO	NO	NO
hsa-miR-188-3p	0.180641994	4339	5068	3111	2044	NO	NO	YES
hsa-miR-1273a	0.182116002	808	1117	2063	418	NO	NO	NO
hsa-miR-1256	0.182240993	2862	5169	3098	1584	NO	NO	YES
hsa-miR-202-5p	0.183076993	-	924	1783	376	NO	NO	NO

hsa-miR-1276	0.183534995	1696	89	3542	-	NO	NO	NO
hsa-miR-135b-3p	0.184383005	-	468	1548	796	NO	NO	YES
hsa-miR-1233-3p	0.187611997	3032	5779	2646	2170	NO	YES	NO
hsa-miR-138-2-3p	0.188162997	-	1420	2946	195	NO	NO	YES
hsa-miR-146a-5p	0.190625995	3200	5519	3004	2531	NO	NO	NO
hsa-miR-374a-3p	0.192799002	-	2745	301	878	NO	NO	NO
hsa-miR-516a-3p	0.194117993	3933	631	-	382	NO	NO	YES
hsa-miR-337-5p	0.200278997	1215	597	6579	242	NO	NO	NO
hsa-miR-589-5p	0.200913996	1610	1612	-	1034	NO	NO	NO
hsa-miR-7-2-3p	0.203990996	-	2814	523	3027	NO	NO	NO
hsa-miR-601	0.205153003	1751	252	-	1104	NO	NO	NO
hsa-miR-196b-5p	0.206462994	2553	1073	-	745	NO	NO	NO
hsa-miR-1246	0.208223999	4379	4822	2229	1318	NO	NO	NO
hsa-miR-376a-3p	0.208574995	964	2500	-	961	NO	NO	NO
hsa-miR-556-5p	0.211430997	376	3003	7444	934	NO	NO	NO
hsa-miR-7-1-3p	0.217892006	-	2921	795	3008	NO	NO	NO
hsa-miR-194-3p	0.218291	-	962	1618	2968	NO	NO	NO
hsa-miR-512-3p	0.221820995	2642	1056	7234	2078	NO	NO	NO
hsa-miR-329-3p	0.223376006	1190	2929	3821	887	NO	NO	YES
hsa-miR-1265	0.224294007	1422	3251	-	871	NO	NO	NO
hsa-miR-1260a	0.231847003	3980	1048	-	395	NO	NO	YES
hsa-miR-448	0.236009002	1055	2441	-	1396	NO	NO	NO
hsa-miR-455-5p	0.238929003	2884	992	-	487	NO	NO	NO
hsa-miR-1227-3p	0.243159994	376	3027	3297	1946	NO	NO	NO
hsa-miR-603	0.245607004	1564	1366	-	2437	NO	NO	NO
hsa-miR-608	0.247343004	535	3021	-	2002	NO	NO	YES
hsa-miR-568	0.248677999	3246	1220	10201	1137	NO	NO	NO
hsa-miR-323b-5p	0.251365006	3727	4042	2608	1623	NO	NO	YES
hsa-miR-186-3p	0.252754003	-	3802	1927	865	NO	NO	NO
hsa-miR-410-3p	0.256489009	569	4546	748	-	NO	NO	NO
hsa-miR-181c-3p	0.258332998	-	2257	1111	565	NO	NO	YES
hsa-miR-885-5p	0.264512002	466	2722	-	1356	NO	NO	YES
hsa-miR-200a-5p	0.266595006	-	1767	1146	1417	NO	NO	NO
hsa-miR-659-3p	0.273077011	913	2847	2642	-	NO	NO	NO
hsa-miR-1825	0.28579399	3399	2005	3594	977	NO	NO	YES
hsa-miR-211-5p	0.286529005	5505	5516	3758	2917	NO	NO	YES
hsa-miR-650	0.28966701	5292	6777	4917	3143	NO	NO	YES
hsa-miR-569	0.290315986	1833	1847	3402	1675	NO	NO	NO
hsa-miR-494-3p	0.290610999	5892	5432	4748	3056	NO	NO	NO
hsa-miR-616-3p	0.293623	3082	2094	4358	858	NO	NO	NO
hsa-miR-301b	0.30015099	658	4054	3776	2259	NO	NO	NO
hsa-miR-301b-3p	0.30015099	658	4054	3776	2259	NO	NO	NO
hsa-miR-506-3p	0.301862001	1868	1884	3914	-	NO	NO	NO

hsa-miR-1226-3p	0.304044992	2658	2606	4910	1474	NO	NO	YES
hsa-miR-656-3p	0.304944009	1723	3782	4577	1475	NO	NO	NO
hsa-miR-513b-5p	0.306062013	560	3803	4368	2499	NO	NO	YES
hsa-miR-637	0.310636014	2855	8294	4878	3705	NO	YES	NO
hsa-miR-15a-3p	0.316477001	-	3300	2374	1259	NO	YES	NO
hsa-miR-491-5p	0.318204999	3395	1740	4864	2894	NO	YES	NO
hsa-miR-182-5p	0.318495005	3105	1525	2643	-	NO	NO	NO
hsa-miR-610	0.318747997	1203	2230	-	778	NO	NO	NO
hsa-miR-150-3p	0.31942901	-	653	3124	1016	NO	NO	NO
hsa-miR-892a	0.323642999	293	3535	651	-	NO	NO	NO
hsa-miR-203a	0.327226996	4226	7392	3448	3763	NO	NO	NO
hsa-miR-203a-3p	0.327226996	4226	7392	3448	3763	NO	NO	NO
hsa-miR-769-5p	0.328711987	1399	2860	-	1889	NO	NO	NO
hsa-miR-454-3p	0.335336	4066	980	-	2303	NO	NO	NO
hsa-miR-371a-5p	0.33723101	5773	504	4939	1971	NO	NO	NO
hsa-miR-218-1-3p	0.343681008	-	1922	2637	877	NO	NO	YES
hsa-miR-548f-3p	0.34453401	2281	2362	-	3487	NO	NO	NO
hsa-miR-591	0.345761001	631	3205	-	912	NO	NO	YES
hsa-miR-95-3p	0.346524	1568	521	-	288	NO	NO	NO
hsa-miR-200b-5p	0.347662985	-	1920	1690	1776	NO	NO	NO
hsa-miR-429	0.350255013	1502	4013	-	1205	NO	NO	YES
hsa-miR-660-5p	0.350504994	1311	2620	2780	1389	NO	NO	NO
hsa-miR-30c-1-3p	0.351233006	-	3410	2446	2899	NO	NO	YES
hsa-miR-378a-5p	0.353208989	-	2598	4124	1060	NO	NO	NO
hsa-miR-222-3p	0.353707999	212	3833	1976	-	NO	NO	YES
hsa-miR-486-3p	0.356628001	4125	1009	16892	2190	NO	NO	NO
hsa-miR-187-5p	0.358808994	-	1409	2121	1865	NO	NO	NO
hsa-let-7a-3p	0.361041009	-	2908	1956	2278	NO	NO	NO
hsa-miR-100-5p	0.362060994	735	863	-	200	NO	NO	NO
hsa-miR-1237-3p	0.364331007	4625	7562	4609	3434	NO	NO	NO
hsa-let-7b-3p	0.366136014	-	2668	2184	2221	NO	NO	NO
hsa-miR-488-3p	0.368782014	390	5955	761	-	NO	NO	NO
hsa-let-7f-1-3p	0.369686991	-	2983	2032	2280	NO	NO	NO
hsa-miR-126-5p	0.371262997	-	2317	3656	2063	NO	NO	NO
hsa-miR-590-5p	0.374002993	1769	4516	-	5152	NO	NO	NO
hsa-miR-877-5p	0.374339998	3103	4385	202	-	NO	NO	YES
hsa-miR-607	0.375542015	1822	1746	-	2198	NO	NO	NO
hsa-miR-548h-5p	0.381455004	2989	4613	-	1677	NO	NO	NO
hsa-miR-500a-3p	0.387724012	-	2322	2499	1246	NO	NO	NO
hsa-miR-1323	0.388114005	3208	3929	4910	1951	NO	NO	NO
hsa-miR-588	0.391874999	2090	3679	-	962	NO	NO	NO
hsa-miR-302f	0.402713001	890	4406	1523	-	NO	NO	NO
hsa-miR-889-3p	0.406574994	1708	5032	5249	943	NO	NO	NO

hsa-miR-155-5p	0.408793002	2540	1535	-	2295	NO	NO	YES
hsa-miR-490-3p	0.413446009	632	4443	3129	2162	NO	NO	NO
hsa-miR-101-5p	0.415030986	-	1968	2411	1624	NO	NO	NO
hsa-miR-145-3p	0.416088998	-	2244	2089	1567	NO	NO	NO
hsa-miR-526b-5p	0.420520008	211	3892	-	2082	NO	NO	YES
hsa-miR-520d-5p	0.426066995	5323	6583	4838	4708	NO	NO	NO
hsa-miR-1224-3p	0.436601013	2796	5367	-	652	NO	NO	YES
hsa-miR-646	0.437252998	4135	3126	4823	2426	NO	YES	NO
hsa-miR-324-3p	0.441781998	1334	4519	3674	-	NO	NO	YES
hsa-miR-148a-3p	0.444471002	3119	3369	5733	2488	NO	NO	YES
hsa-miR-760	0.444826007	2756	5572	-	759	NO	NO	YES
hsa-miR-148b-3p	0.450354993	3208	3382	11119	2489	NO	NO	YES
hsa-miR-1179	0.451671004	3006	3343	-	2315	NO	NO	NO
hsa-miR-450b-5p	0.454584986	2039	5058	17036	3078	NO	NO	NO
hsa-miR-548o-3p	0.458454013	3624	4236	4403	2191	NO	NO	NO
hsa-miR-520c-3p	0.459800988	1945	5410	17257	2441	NO	NO	YES
hsa-miR-30a-3p	0.467294008	-	5763	1463	3131	NO	NO	NO
hsa-miR-634	0.467777014	3142	2964	16300	2294	NO	NO	YES
hsa-miR-302b-3p	0.471944004	2092	5542	11732	2380	NO	NO	NO
hsa-miR-33a-3p	0.475270003	-	1423	3436	3293	NO	NO	YES
hsa-miR-617	0.475704998	4147	2173	2583	1513	NO	NO	NO
hsa-miR-541-3p	0.476085007	2284	3297	5274	-	NO	NO	NO
hsa-miR-338-3p	0.480908006	4369	2589	3671	-	NO	NO	YES
hsa-miR-497-3p	0.484751999	-	6703	1186	1920	NO	NO	NO
hsa-miR-576-5p	0.488083005	1234	6065	4386	2300	NO	NO	NO
hsa-miR-148b-5p	0.49287799	-	4292	3172	1340	NO	NO	NO
hsa-miR-221-5p	0.494044989	-	6142	2691	1908	NO	NO	NO
hsa-miR-125b-2-3p	0.498228997	-	3488	3246	2288	NO	NO	YES
hsa-miR-302e	0.499166995	2839	4983	17224	2339	NO	NO	YES
hsa-miR-520d-3p	0.501421988	2644	5409	17209	2386	NO	NO	NO
hsa-miR-31-3p	0.502478004	-	3169	1547	982	NO	NO	NO
hsa-miR-302c-3p	0.504777014	2250	5530	8479	3361	NO	NO	YES
hsa-miR-30c-2-3p	0.507638991	-	3313	5365	2787	NO	NO	YES
hsa-miR-875-3p	0.508500993	4825	4069	6307	3227	NO	NO	NO
hsa-miR-520b	0.509263992	2695	5350	17259	2440	NO	NO	YES
hsa-miR-570-3p	0.51017803	4501	6058	5064	1660	NO	NO	NO
hsa-miR-520e	0.516013026	2911	5323	17232	2344	NO	NO	YES
hsa-miR-302a-3p	0.516183972	2801	5520	17193	2374	NO	NO	NO
hsa-miR-141-3p	0.521520972	2580	4029	4074	2439	NO	NO	YES
hsa-miR-373-3p	0.525241017	3495	5623	17268	1826	NO	NO	NO
hsa-miR-330-5p	0.531329989	2070	3163	2788	-	NO	YES	NO
hsa-miR-19b-3p	0.532522976	2579	4052	16127	2816	NO	NO	NO
hsa-miR-27a-5p	0.535273015	-	4714	2690	727	NO	NO	NO

hsa-miR-15b-5p	0.535530984	1111	7497	3124	-	NO	NO	NO
hsa-miR-1238-3p	0.536861002	3817	3577	3359	-	NO	NO	YES
hsa-miR-3188	0.538332999	-	329	-	203	NO	NO	NO
hsa-miR-320d	0.540476024	3947	4301	7370	2597	NO	NO	NO
hsa-miR-623	0.547326028	4892	5111	4617	1305	NO	YES	NO
hsa-miR-579-3p	0.550613999	5135	5436	4825	2104	NO	NO	NO
hsa-miR-7-5p	0.556505978	2324	5985	4568	-	NO	NO	NO
hsa-miR-548s	0.556568027	-	559	-	311	NO	NO	YES
hsa-miR-200a-3p	0.558322012	3138	3911	4332	2410	NO	NO	YES
hsa-miR-1183	0.561589003	428	6402	-	2500	NO	NO	NO
hsa-miR-638	0.564881027	2606	1011	4303	-	NO	NO	NO
hsa-miR-320c	0.565195024	4293	4359	8102	2612	NO	NO	NO
hsa-miR-580-3p	0.568499029	4123	6351	4867	2139	NO	NO	NO
hsa-miR-130b-5p	0.569827974	-	7487	185	2896	YES	NO	YES
hsa-miR-1202	0.571497977	3109	4406	-	1886	NO	NO	NO
hsa-miR-26a-1-3p	0.579515994	-	509	7103	341	NO	NO	NO
hsa-miR-520a-3p	0.580022991	3834	5249	17230	2386	NO	NO	NO
hsa-miR-516b-5p	0.585080981	4649	5473	-	2213	NO	NO	NO
hsa-miR-26a-2-3p	0.585433006	-	578	5461	344	NO	NO	NO
hsa-miR-221-3p	0.588545978	1219	4077	2946	-	NO	YES	NO
hsa-miR-324-5p	0.593029022	1814	4129	2383	-	NO	NO	NO
hsa-miR-21-3p	0.593097985	-	4656	2295	1488	NO	NO	YES
hsa-miR-587	0.594022989	1082	6938	-	3383	NO	NO	YES
hsa-miR-641	0.59455198	2374	6243	2499	-	NO	NO	NO
hsa-miR-34a-3p	0.59621799	-	4804	2535	2065	NO	NO	NO
hsa-miR-519e-5p	0.600230992	-	8100	2554	1689	NO	NO	YES
hsa-miR-320a	0.601840973	4859	4447	13489	2606	YES	NO	YES
hsa-miR-320b	0.603555024	4884	4447	12623	2628	NO	NO	YES
hsa-miR-508-3p	0.609637022	3233	3686	1766	-	NO	NO	YES
hsa-miR-888-3p	0.610174	-	5065	2019	951	NO	NO	YES
hsa-miR-372-3p	0.618840992	4274	5381	17131	2464	NO	NO	NO
hsa-miR-562	0.626774013	3219	5552	3387	2135	NO	NO	NO
hsa-miR-29a-5p	0.633677006	-	6454	355	2942	NO	NO	NO
hsa-miR-302b-5p	0.636159003	-	6918	3007	2028	NO	NO	NO
hsa-miR-149-5p	0.637017012	2416	6435	3973	-	NO	YES	NO
hsa-miR-1285-3p	0.638437986	3260	7417	4019	-	NO	NO	NO
hsa-miR-532-5p	0.64130199	1129	779	16961	-	NO	NO	YES
hsa-miR-1257	0.641606987	3175	3828	3298	-	NO	NO	NO
hsa-miR-19a-3p	0.64240402	3678	4171	-	2814	NO	NO	NO
hsa-miR-204-5p	0.644636989	5090	5540	4723	2313	NO	NO	YES
hsa-miR-1297	0.644662976	4967	3064	3781	-	NO	NO	NO
hsa-miR-22-5p	0.647931993	-	5221	2671	2670	NO	NO	NO
hsa-miR-612	0.648656011	2362	7732	3988	-	NO	YES	NO

hsa-miR-130a-5p	0.651410997	-	4723	3506	3320	NO	YES	NO
hsa-miR-370-3p	0.652806997	5342	5008	11266	2605	NO	NO	NO
hsa-miR-125a-5p	0.65437901	3236	4700	3427	-	NO	NO	NO
hsa-miR-1207-5p	0.659171999	5043	4194	6741	-	NO	NO	YES
hsa-miR-885-3p	0.666885972	3455	5601	-	2139	NO	NO	YES
hsa-miR-1298-5p	0.669445992	1290	5528	2854	-	NO	NO	NO
hsa-miR-922	0.678541005	1961	7165	4562	-	NO	NO	NO
hsa-miR-106b-3p	0.678686976	-	528	1359	223	NO	NO	YES
hsa-miR-671-5p	0.680431008	2667	3705	5015	-	NO	NO	YES
hsa-miR-25-3p	0.690807998	4065	5423	1741	-	NO	NO	NO
hsa-miR-302a-5p	0.691338003	-	1058	7211	1807	NO	NO	NO
hsa-miR-21-5p	0.692276001	944	1236	16804	-	NO	NO	NO
hsa-miR-214-3p	0.692282021	1318	2282	11561	-	NO	NO	NO
hsa-miR-1248	0.693575978	3458	7863	4089	3196	NO	NO	NO
hsa-miR-302d-5p	0.695556998	-	6936	2389	1925	NO	NO	NO
hsa-miR-493-5p	0.699221015	-	5294	3265	2776	NO	NO	YES
hsa-miR-513c-5p	0.703764021	5711	6003	4838	2356	NO	NO	YES
hsa-miR-382-5p	0.705784023	2052	888	16914	-	NO	NO	YES
hsa-miR-1253	0.706465006	5599	6826	3773	-	NO	NO	NO
hsa-miR-939-5p	0.710116982	5894	5089	-	3147	NO	YES	NO
hsa-miR-1258	0.713051975	5442	3785	2844	1329	NO	NO	NO
hsa-miR-384	0.724189997	479	2762	3468	-	NO	NO	NO
hsa-miR-124-5p	0.725263	-	4952	2585	1964	NO	NO	YES
hsa-miR-92a-3p	0.725795984	4064	5345	2394	-	NO	YES	NO
hsa-miR-127-5p	0.729249001	3415	5740	3740	-	NO	NO	YES
hsa-miR-488-5p	0.736375988	-	1312	16999	1674	NO	NO	NO
hsa-miR-373-5p	0.737739027	-	2605	5009	1822	NO	NO	YES
hsa-miR-1231	0.744714975	5415	6332	2176	-	NO	NO	YES
hsa-miR-551b-5p	0.761790991	-	3510	4643	1115	NO	NO	NO
hsa-miR-548l	0.767163992	5031	7765	5295	2610	NO	NO	NO
hsa-miR-138-1-3p	0.770226002	-	2114	2598	873	NO	NO	NO
hsa-miR-629-3p	0.770277977	-	7994	4613	2734	NO	NO	YES
hsa-miR-548e-3p	0.77226001	2928	7418	-	3440	NO	NO	YES
hsa-miR-335-3p	0.774380982	-	3820	6865	1583	NO	NO	NO
hsa-miR-185-3p	0.775705993	-	2126	11902	1782	NO	YES	NO
hsa-miR-654-5p	0.778514981	1954	3171	7598	-	NO	NO	NO
hsa-miR-544a	0.783111989	6393	6774	3211	-	NO	NO	NO
hsa-miR-34c-5p	0.791676998	3942	790	6053	-	NO	NO	NO
hsa-miR-411-3p	0.792636991	-	2540	2691	1147	NO	NO	NO
hsa-miR-619-3p	0.794385016	2190	2263	8814	-	NO	NO	NO
hsa-miR-518e-3p	0.796905994	1576	340	16814	-	NO	NO	NO
hsa-miR-573	0.800228	5307	5834	3939	-	NO	NO	NO
hsa-miR-379-3p	0.800768018	-	2708	2999	1151	NO	NO	NO

hsa-miR-133a-3p	0.800979018	126	4156	4377	-	NO	NO	YES
hsa-miR-34a-5p	0.801451981	4243	727	6563	-	NO	NO	NO
hsa-miR-337-3p	0.809539974	398	3417	16491	-	NO	NO	YES
hsa-miR-576-3p	0.818086982	4104	792	2907	-	NO	NO	NO
hsa-miR-33b-5p	0.818750024	3717	1143	6535	-	NO	NO	YES
hsa-miR-133b	0.822797	246	4156	4703	-	NO	NO	YES
hsa-miR-1295a	0.824312985	208	2507	15073	-	NO	NO	YES
hsa-miR-888-5p	0.825070977	3715	946	15575	-	NO	NO	NO
hsa-miR-34b-3p	0.831609011	1915	2629	6150	-	NO	NO	YES
hsa-miR-30b-3p	0.832422018	-	7707	5742	4874	NO	NO	NO
hsa-miR-1263	0.834183991	1598	1758	-	-	NO	NO	NO
hsa-miR-1279	0.841153026	651	4644	3104	-	NO	NO	NO
hsa-miR-10b-5p	0.842842996	4022	6407	-	2452	NO	NO	NO
hsa-miR-627-5p	0.843616009	1006	4017	4298	-	NO	NO	YES
hsa-miR-1283	0.843748987	915	4521	3582	-	NO	NO	NO
hsa-miR-10a-5p	0.845750988	4105	6470	-	2457	NO	NO	YES
hsa-miR-1225-3p	0.848917007	4491	5508	-	2502	NO	YES	NO
hsa-miR-508-5p	0.849784017	1886	4517	5370	-	NO	NO	YES
hsa-miR-331-5p	0.852726996	1017	3446	3902	-	NO	NO	YES
hsa-miR-507	0.853317976	4517	1648	13071	-	NO	NO	YES
hsa-miR-630	0.862909973	1643	3354	8094	-	NO	NO	YES
hsa-miR-548n	0.863903999	604	6180	6049	-	NO	NO	NO
hsa-miR-708-5p	0.865096986	4390	2146	9528	-	NO	NO	YES
hsa-miR-519c-3p	0.86897099	3457	3008	15230	-	NO	NO	YES
hsa-miR-543	0.870742977	1652	5169	4572	-	NO	NO	YES
hsa-miR-26b-5p	0.874917984	3015	3537	4321	-	NO	NO	NO
hsa-miR-1224-5p	0.875396013	3432	3677	4471	-	NO	NO	YES
hsa-miR-363-5p	0.877811015	-	3652	10735	2959	NO	NO	YES
hsa-miR-302c-5p	0.878795981	-	4055	4211	2021	NO	NO	YES
hsa-miR-129-2-3p	0.879392982	3998	2211	3677	-	NO	NO	NO
hsa-miR-935	0.881386995	2289	3793	4024	-	NO	YES	NO
hsa-miR-153-3p	0.887670994	1185	4557	4318	-	NO	NO	YES
hsa-miR-323a-3p	0.892503977	5040	1142	7143	-	NO	NO	NO
hsa-miR-642a-5p	0.893747985	3784	3372	12311	-	NO	NO	NO
hsa-miR-411-5p	0.895319998	722	5829	2311	-	NO	NO	NO
hsa-miR-1269a	0.896767974	1977	2606	6137	-	NO	NO	YES
hsa-miR-632	0.898676991	873	6370	5085	-	NO	NO	NO
hsa-miR-323a-5p	0.898778975	1297	5597	5851	-	NO	YES	NO
hsa-miR-331-3p	0.898838997	3058	3659	5215	-	NO	NO	NO
hsa-miR-647	0.900600016	909	6809	5550	-	NO	NO	NO
hsa-miR-216a-5p	0.901630998	2436	4586	5325	-	NO	NO	NO
hsa-miR-181c-5p	0.902131975	4412	2955	8892	-	NO	NO	YES
hsa-miR-631	0.90417999	985	4867	6056	-	NO	NO	NO

hsa-miR-326	0.904811025	1492	3046	5312	-	NO	YES	NO
hsa-miR-125b-5p	0.910080016	2593	4633	16395	-	NO	NO	YES
hsa-miR-215-5p	0.910381973	1372	5046	5735	-	NO	NO	NO
hsa-miR-875-5p	0.910911024	862	4105	2269	-	NO	NO	NO
hsa-miR-22-3p	0.911913991	850	5743	3756	-	NO	NO	NO
hsa-miR-16-5p	0.913622022	1337	7522	5233	-	NO	NO	YES
hsa-miR-513a-3p	0.914426029	274	8689	5064	-	NO	NO	NO
hsa-miR-193a-3p	0.916751027	1666	4326	4192	-	NO	NO	NO
hsa-miR-28-5p	0.917303979	6183	2034	8069	-	NO	NO	NO
hsa-miR-26a-5p	0.917753994	4514	3391	4148	-	NO	NO	NO
hsa-miR-577	0.921604991	1790	6340	4555	-	NO	NO	NO
hsa-let-7g-3p	0.922379017	-	4929	3689	2051	NO	YES	NO
hsa-miR-219a-5p	0.926090002	2264	3160	15769	-	NO	NO	NO
hsa-miR-664a-3p	0.926340997	1573	6886	8538	-	NO	NO	NO
hsa-miR-501-3p	0.928975999	1116	2074	2673	-	NO	NO	NO
hsa-miR-192-5p	0.929176986	2034	5046	4916	-	NO	NO	NO
hsa-miR-649	0.930019021	1370	5440	3132	-	NO	NO	NO
hsa-miR-616-5p	0.930212021	-	5867	4718	2138	NO	NO	YES
hsa-miR-380-3p	0.930253983	1471	6307	3701	-	NO	NO	NO
hsa-miR-140-3p	0.932790995	2918	5917	3634	-	NO	NO	NO
hsa-miR-92b-3p	0.933541	2319	5293	3282	-	NO	NO	NO
hsa-miR-342-3p	0.935725987	2244	6339	3830	-	NO	NO	NO
hsa-miR-890	0.93699801	2322	6290	6434	-	NO	NO	NO
hsa-miR-497-5p	0.940972984	2660	7459	16313	-	NO	NO	YES
hsa-miR-1225-5p	0.941091001	4495	2485	5280	-	NO	NO	NO
hsa-miR-520f-3p	0.945172012	2273	4554	7070	-	NO	NO	YES
hsa-miR-93-3p	0.945330024	-	6734	13971	3137	NO	NO	YES
hsa-miR-502-3p	0.945379972	1640	2247	4217	-	NO	NO	NO
hsa-miR-363-3p	0.945716977	2600	5386	3285	-	NO	NO	NO
hsa-miR-30a-5p	0.947404027	2451	5799	16478	-	NO	NO	NO
hsa-miR-519b-3p	0.948282003	2938	6539	17068	-	NO	NO	NO
hsa-miR-339-5p	0.952144027	3428	5991	10461	-	NO	NO	YES
hsa-miR-30b-5p	0.954083979	2759	5797	6113	-	NO	NO	NO
hsa-miR-1322	0.955558002	3585	3436	3280	-	NO	NO	YES
hsa-miR-425-5p	0.95590198	4549	4032	16526	-	NO	NO	NO
hsa-miR-15a-5p	0.956933975	3500	7523	8657	-	NO	NO	NO
hsa-miR-340-5p	0.961197019	3603	6675	4761	-	NO	NO	NO
hsa-miR-375	0.961601973	2933	4887	7818	-	NO	NO	YES
hsa-miR-502-5p	0.961920023	5290	5274	9461	-	NO	NO	YES
hsa-miR-16-2-3p	0.962795019	-	6152	3564	2660	NO	NO	NO
hsa-miR-452-5p	0.965198994	4512	5641	3608	-	NO	NO	NO
hsa-miR-30d-5p	0.965260983	3464	5697	15529	-	NO	NO	NO
hsa-miR-1254	0.966180027	5711	7156	16777	-	NO	NO	NO

hsa-miR-30e-5p	0.967535973	3590	5897	15903	-	NO	NO	NO
hsa-miR-30c-5p	0.968336999	3606	5821	14819	-	NO	NO	NO
hsa-miR-32-5p	0.971656978	4045	5533	3312	-	NO	NO	NO
hsa-miR-514a-3p	0.974066973	4240	4996	14145	-	NO	NO	NO
hsa-miR-622	0.977292001	5132	6845	4811	-	NO	NO	NO
hsa-miR-1200	0.977927029	5278	7463	16907	-	NO	NO	NO
hsa-miR-512-5p	0.979613006	4728	4104	16622	-	NO	NO	YES
hsa-miR-557	0.980004013	5334	6952	5192	-	NO	NO	NO
hsa-miR-205-5p	0.980677009	4651	7236	4749	-	NO	NO	NO
hsa-miR-876-3p	0.980974972	5899	5759	6960	-	NO	NO	NO
hsa-miR-367-3p	0.98160398	4987	5453	3376	-	NO	NO	NO
hsa-miR-668-3p	0.983757019	5025	5535	16622	-	NO	YES	NO
hsa-miR-1207-3p	0.984812021	4800	8654	2873	-	NO	NO	YES
hsa-miR-575	0.985710025	5777	8007	11921	-	NO	YES	NO
hsa-miR-499a-3p	0.987583995	3632	4144	8926	-	NO	NO	NO
