# Supplementary material for: miR-514a-3p: a novel SHP-2 regulatory miRNA that modulates human cytotrophoblast proliferation
Source: J Mol Endocrinol. 2021 Nov 18;68(2):99–110. doi: 10.1530/JME-21-0175 (PMC8789026; doi:10.1530/JME-21-0175)
Supplement: Supplementary Table 3 [file supplementary_table_3.pdf]

miRNA	Fold change in PTPN11 expression (relative to 18SrNA)	Predicted to target *PTPN11*
hsa-miR-574-5p	1.64	NO
hsa-miR-184	1.28	NO
**hsa-miR-660**	**1.17**	**YES**
**hsa-miR-337-5p**	**1**	**YES**
**hsa-miR-146b-5p**	**1.36**	**YES**
hsa-miR-432	1.31	NO
**hsa-miR-370**	**1.23**	**YES**
**hsa-miR-95-3p**	**-1.08**	**YES**
hsa-miR-206	1.27	NO
**hsa-miR-873-5p**	**1.26**	**YES**
hsa-miR-532-3p	-1.01	NO
hsa-miR-139-3p	1.76	NO
**hsa-miR-524-5p**	**1.07**	**YES**
**hsa-miR-188-3p**	**-1.01**	**YES**
**hsa-miR-590-5p**	**1.08**	**YES**
**hsa-miR-455-5p**	**1.06**	**YES**
**hsa-miR-331-5p**	**-1.1**	**YES**
**hsa-miR-429**	**-1.25**	**YES**
**hsa-miR-362-5p**	**1.54**	**YES**
hsa-miR-525-3p	1.26	NO
**hsa-miR-493**	**1.09**	**YES**
**hsa-miR-526b**	**-1.24**	**YES**
hsa-miR-190	1.27	NO
**hsa-miR-515-5p**	**1.77**	**YES**
hsa-miR-665	1.23	NO
hsa-miR-147a	-1.42	NO
**hsa-miR-361-3p**	-1.06	YES
**hsa-miR-514**	**-3.4**	**YES**
**hsa-miR-376b**	**-1.25**	**YES**
**hsa-miR-582-5p**	**1.45**	**YES**
**hsa-miR-187-3p**	**1.11**	**YES**
**hsa-miR-671-5p**	**1.1**	**YES**
hsa-miR-487b	-1.12	NO
**hsa-miR-651**	**-1.06**	**YES**
**hsa-miR-219a-5p**	**1.28**	**YES**
hsa-miR-299-3p	-1.03	NO
hsa-miR-449a	1.23	NO
**hsa-miR-369-3p**	**1.16**	**YES**
hsa-miR-219-2-3p	1.28	NO
**hsa-miR-105-5p**	**-1.07**	**YES**

**Key**
Increased >2 fold
Decreased>2fold
**Predicted to target PTPN11 in bold**

hsa-miR-551b-3p	-1.12	NO
hsa-miR-542-5p	1.14	NO
**hsa-miR-542-3p**	**1.24**	**YES**
**hsa-miR-618**	**-1.35**	**YES**
**hsa-miR-502-3p**	**-1.08**	**YES**
hsa-miR-519a-3p	-1.14	NO
hsa-miR-181d	1.15	NO
**hsa-miR-382-5p**	**1.33**	**YES**
**hsa-miR-508-3p**	**1.28**	**YES**
hsa-miR-450a	1.08	NO
**hsa-miR-127-5p**	**1.07**	**YES**
hsa-miR-523	1.1	NO
**hsa-miR-589**	**1.87**	**YES**
hsa-miR-369-5p	1.29	NO
hsa-miR-615-3p	1.17	NO
**hsa-miR-518a-5p**	**-1.19**	**YES**
**hsa-miR-576-3p**	**1.12**	**YES**
**hsa-miR-337-3p**	**1.06**	**YES**
**hsa-miR-362-3p**	**1.07**	**YES**
hsa-miR-515-3p	1.53	NO
hsa-miR-518a-3p	1.32	NO
**hsa-miR-885-5p**	**1.86**	**YES**
hsa-miR-433	1.3	NO
**hsa-miR-512-3p**	**1.58**	**YES**
**hsa-miR-548d-5p**	**1.59**	**YES**
**hsa-miR-133b**	**1.74**	**YES**
hsa-miR-296-5p	1.13	NO
hsa-miR-371a-3p	-1.15	NO
**hsa-miR-330-3p**	**-1.31**	**YES**
**hsa-miR-329**	**-1.44**	**YES**
**hsa-miR-495-3p**	**1.19**	**YES**
**hsa-miR-450b-5p**	**1.46**	**YES**
**hsa-miR-758-3p**	**2.16**	**YES**
**hsa-miR-522-3p**	**-1.04**	**YES**
**hsa-miR-323a-3p**	**1.3**	**YES**
**hsa-miR-889**	**1.25**	**YES**
**hsa-miR-208b**	**1.11**	**YES**
hsa-miR-487a	-1.06	NO
hsa-miR-628-3p	-1.12	NO
**hsa-miR-628-5p**	**-1.12**	**YES**
hsa-miR-671-3p	1.45	NO
hsa-miR-485-3p	-1.15	NO
**hsa-miR-548d-3p**	**1.29**	**YES**

**hsa-miR-383**	**1.62**	**YES**
**hsa-miR-548b-3p**	**1.07**	**YES**
**hsa-miR-548n**	**1.27**	**YES**
**hsa-miR-573**	**-1.14**	**YES**
**hsa-miR-587**	**-1.01**	**YES**
**hsa-miR-621**	**-1.39**	**YES**
hsa-miR-629-5p	-1.39	NO
